# Type I Interferon Drives a Cellular State Inert to TCR‐Stimulation and Could Impede Effective T‐Cell Differentiation in Cancer

**DOI:** 10.1002/eji.202451371

**Published:** 2024-11-12

**Authors:** Dillon Corvino, Martin Batstone, Brett G. M Hughes, Tim Kempchen, Susanna S Ng, Nazhifah Salim, Franziska Schneppenheim, Denise Rommel, Ananthi Kumar, Sally Pearson, Jason Madore, Lambross T. Koufariotis, Lisa Maria Steinheuer, Dilan Pathirana, Kevin Thurley, Michael Hölzel, Nicholas Borcherding, Matthias Braun, Tobias Bald

**Affiliations:** ^1^ Tumor‐Immunobiology Institute for Experimental Oncology University Hospital Bonn Bonn Germany; ^2^ Royal Brisbane and Women's Hospital Brisbane Queensland Australia; ^3^ Faculty of Medicine University of Queensland Brisbane Queensland Australia; ^4^ QIMR Berghofer Medical Research Institute Herston Australia; ^5^ Faculty of Mathematics and Natural Sciences and the Life and Medical Sciences Institute (LIMES) Rheinische Friedrich‐Wilhelms‐Universität Bonn Bonn Germany; ^6^ Department of Pathology and Immunology Washington University School of Medicine St. Louis Missouri USA; ^7^ Department of Pediatric Hematology, Oncology and Immunodeficiency University Childrens Hospital of the Justus‐Liebig University Gießen Gießen Germany

**Keywords:** CD8^+^ T‐cells, HNSCC TILs, scRNAseq, scTCRseq, Type I IFN

## Abstract

**Background:**

Head and neck squamous cell carcinoma (HNSCC) is linked to human papillomavirus (HPV) infection. HPV‐positive and HPV‐negative HNSCC exhibit distinct molecular and clinical characteristics. Although checkpoint inhibitors have shown efficiency in recurrent/metastatic HNSCC, response variability persists regardless of HPV status. This study aimed to explore the CD8^+^ T‐cell landscape in HPV‐negative HNSCC.

**Methods:**

We performed simultaneous single‐cell RNA and TCR sequencing of CD8^+^ tumor‐infiltrating lymphocytes (TILs) from treatment‐naïve HPV‐negative HNSCC patients. Additionally, cells were stimulated ex vivo, which allowed for the tracking of clonal transcriptomic responses.

**Results:**

Our analysis identified a subset of CD8^+^ TILs highly enriched for interferon‐stimulated genes (ISG). TCR analysis revealed ISG cells are clonally related to a population of granzyme K (GZMK)‐expressing cells. However, unlike GZMK cells, which exhibited rapid effector‐like phenotypes following stimulation, ISG cells were transcriptionally inert. Additionally, ISG cells showed specific enrichment within tumor and were found across multiple tumor entities.

**Conclusions:**

ISG‐enriched CD8^+^ TILs are a consistent feature of various tumor entities. These cells are poorly understood but possess characteristics that may impact antitumor immunity. Understanding the unique properties and functionality of ISG cells could offer innovative treatment approaches to improve patient outcomes in HPV‐negative HNSCC and other cancer types.

## Graphical abstract

Multimodal single‐cell RNA sequencing identified a population of CD8+ T cells elevated in tumor tissue enriched for interferon‐stimulated genes (ISG). These ISG cells are clonally related to a population of Granzyme K (GZMK)‐expressing CD8^+^ T cells. However, unlike GZMK‐expressing T cells, ISG cells were transcriptionally inert in response to in vitro stimulation. Created in BioRender.com

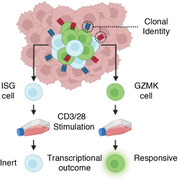

## 1 Introduction

Head and neck squamous cell carcinoma (HNSCC) encompasses cancers originating from the mucosal epithelium of the oral cavity, pharynx, or larynx. HNSCC is closely associated with myriad environmental and lifestyle factors such as air pollutants, tobacco, and alcohol consumption [1]. In addition, viral co‐infection with human papillomavirus (HPV) is observed in a subset of HNSCC (∼32%) patients [2]. Interestingly, HPV‐positive HNSCC is associated with a more favorable prognosis, especially in early‐stage disease [3, 4, 5]. The clinical benefit of HPV status is thought to derive from HPV‐specific immune responses and the intrinsic immunogenicity of HPV [6, 7].

Standard‐of‐care treatment options for HNSCC include surgical resection, radiotherapy, and chemotherapy [1]. However, immunotherapy‐based treatment approaches such as immune checkpoint inhibition (ICI), have shown significant clinical benefit in the recurrent/metastatic setting [8]. In fact, immune checkpoint inhibition has been approved for first‐line treatment of patients with recurrent/metastatic (R/M) HNSCC [9]. Unfortunately, the response to immunotherapy varies significantly. Variable responses may, in part, be attributed to the immunosuppressive tumor‐microenvironment (TME) commonly observed in HNSCC [1]. While it is generally accepted that HPV‐positive HNSCC shows more robust antitumor immune responses compared with HPV‐negative HNSCC, recent immunotherapy trials did not find an association between HPV status and response [10, 11]. Given, that CD8^+^ T‐cells are recognized as key drivers of antitumoral responses, a better understanding of the CD8^+^ tumor‐infiltrating lymphocyte (TIL) heterogeneity in HPV‐negative patients is needed to improve the treatment for this subgroup of HNSCC.

Interferons (IFNs) are pleiotropic cytokines primarily produced by immune and stromal cells in response to pathogens or malignant transformation. Three types of IFNs have been described, which differ by the distinct receptors they bind and the subsequent signaling cascades induced. Type I IFNs (IFN‐I) have well‐described roles in both anti‐viral and antitumor responses. In particular, IFN‐I can directly inhibit tumor growth by inhibiting proliferation and inducing apoptosis. In addition, IFN‐I can act indirectly to induce antitumor immune responses, for example, via the activation of dendritic cells, natural killer cells, or neutrophils [12]. Simultaneously, IFN‐I can reduce the protumorigenic functions of regulatory T‐cells and myeloid‐derived suppressor cells [13]. In fact, IFN‐I signaling is considered a “third signal” of activation and important for naïve T‐cell priming, activation, proliferation, and memory differentiation [14]. Thus, IFN‐I is regarded as a crucial cytokine in facilitating cancer immunosurveillance and boosting the efficacy of cancer immunotherapies [13, 15–17]. However, we have previously shown via genetic ablation, that IFN‐I signaling is dispensable for the expansion and function of adoptively transferred tumor‐specific CD8+ T‐cells [17]. In addition, several studies also provide evidence that IFN‐I signaling, at least in the later stages of antitumor immune responses, can promote protumor changes and ultimately immune escape [18]. For example, IFN‐I signaling is linked to the expression of immune checkpoints, IL‐10, Nos2, and the development of a T‐cell exhaustion phenotype [17, 19, 20]. Therefore, the effect of IFN‐I signaling on the functional outcomes of tumor‐infiltrating T‐cells is multifaceted and requires further investigation.

Single‐cell RNA sequencing (scRNA‐seq) of immune cell subsets in cancer patients has enabled the high‐resolution mapping of cellular heterogeneity. This methodology has been applied to the analysis of human T‐cells in response to cancer immunotherapies [21]. However, traditionally this approach only focuses on assessing the transcriptional state of *ex vivo* isolated cells. Thus, capturing a snapshot of the cellular transcriptomic landscape within the TME. Therefore, we leveraged an ex vivo perturbation via a short‐term T‐cell receptor (TCR) stimulation. Coupled with scRNAseq and single‐cell TCR sequencing, we were able to study the clonal dynamics and evaluate the responsive potential of CD8^+^ TIL subsets.

Herein, we sequenced over 11,000 resting and stimulated CD8^+^ TILs isolated from treatment‐naïve HPV‐negative HNSCC patients. As such, we were able to define ex vivo cellular states and their stimulation outcomes. Importantly, we identified a population of T‐cells rich in IFN‐stimulated genes (ISG). These ISG cells were found to be associated with an IFN‐I signature and were specifically enriched within the tumor tissue of various tumor entities. Furthermore, these cells were found to be clonally related to a population of cells highly expressing granzyme K (GzmK). However, unlike the GzmK subset, ISG‐cells were transcriptionally inert to stimulation and thus possibly possess a unique role within the TME. This study sheds light on the existence of this overlooked population and begins to investigate their functionality.

## 2 Results

### 2.1 Single‐cell RNA Sequencing of CD8^+^ TILs from Treatment‐naive HNSCC Patients Identifies Exhausted and Effector Populations

**FIGURE 1:**
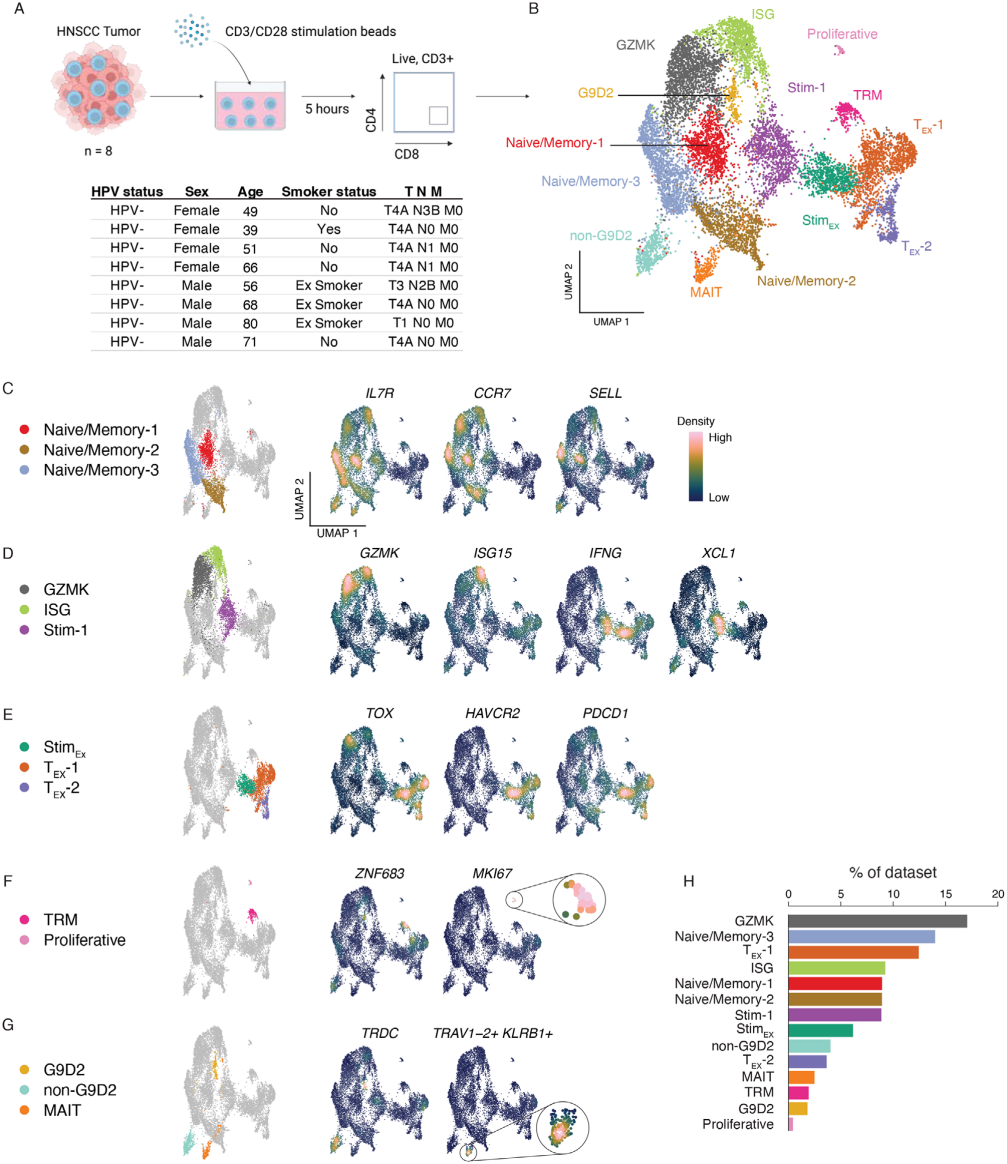
The transcriptional landscape of tumor‐infiltrating CD8+ TILs in treatment‐naive in head and neck squamous cell carcinoma (HNSCC) patients. (A) schematic detailing the experimental setup used to generate the dataset created in BioRender.com. In brief, the tumors from eight head and neck squamous cell carcinoma (HNSCC) patients were digested and processed into a single‐cell suspension. The cell suspension was cultured for 5 h with or without CD3/CD28 T‐cell stimulation. Subsequently, the cells were sorted for CD3^+^CD4‐CD8^+^ T‐cells and subjected to 10× single‐cell sequencing. Key patient characteristics are listed in the table below the schematic. All patients were HPV negative, treatment naïve, and samples were from primary tumors. Schematic created with BioRender.com (B) UMAP projection of all cells that passed QC inclusion criteria. (C–G) UMAP projections highlighting (first column) clusters identified and subsequently the expression density of key genes used in their identification. (G) MAIT‐cell identity is highlighted using the joint density expression of TRAV1‐2 and KLRB1 (H) Barplot showing the frequency of each cluster identified as a proportion of the entire dataset. Data representative of eight patients acquired in one sequencing experiment.

CD8^+^ T‐cells are key drivers of antitumor responses. However, there is substantial heterogeneity in CD8^+^ T‐cell phenotypes within TIL populations. As such, we sought to explore the diversity of CD8^+^ TILs in HPV‐negative treatment‐naïve non‐R/M HNSCC patients. We isolated live CD45^+^CD3^+^CD4‐CD8^+^ from eight patients using flow cytometry‐based cell sorting and subjected half of those cells to *ex vivo* CD3/28 TCR stimulation. After 5 h of stimulation, we performed single‐cell RNA and TCR sequencing to simultaneously identify CD8^+^ TIL phenotypes and clonotypes. We thereby were able to profile transcriptional changes in response to TCR‐based stimulation (Figure 1A).

Sequencing data from both unstimulated and TCR‐stimulated samples were integrated and projected onto a unified UMAP space (Figure 1B). This resulted in 14 distinct clusters of CD8^+^ TILs with the majority of identified clusters evenly distributed across both unstimulated and stimulated conditions (Figure S1A). Importantly, two new clusters emerged specifically post‐TCR‐stimulation (clusters Stimulated‐1; Stim‐1 and Stimulated‐Exhausted; Stim_EX_). Three naïve/memory cell clusters were identified and annotated based on their expression of markers such as *IL7R*, *CCR7*, and *SELL* (Figure 1C). A cluster of cells expressing *GZMK* as well as *EOMES*, *NKG7*, *TNFRSF18* (encodes for GITR), and *CD69* was also identified (Figure 1D and data not shown). Additionally, a cluster of cells expressing high levels of various interferon‐stimulated genes, including *ISG15*, *IFI6*, *IFIT3*, *MX1*, *ISG20*, *IFITM1*, *IFIT1*, *MX2*, and *OAS3* (Figure S1B and data not shown) was recognized and annotated as the interferon‐gene stimulated (ISG) cluster of cells (Figure 1D). The stimulated‐1 (Stim‐1) cluster from TCR‐stimulated cells was enriched for the expression of immune effector molecules such as *IFNG*, *XCL1*, *XCL2*, *CRTAM*, *TNF*, *TNFSF14* (encodes for LIGHT) and *TNFRSF9* (encodes for 4‐1BB) (Figure 1D and Figure S1B). Three exhausted cell clusters were also identified, all expressing high levels of canonical exhaustion markers such as *TOX*, *HAVCR2*, *PDCD1* (encodes for Tim‐3 and PD‐1, respectively), *CTLA4*, *ENTPD1* (encodes CD39), and *TIGIT* (Figure 1E and Figure S1B). One of these exhausted clusters was exclusively found post‐TCR‐stimulation and as such was designated as the stimulated‐exhausted (Stim_EX_) cluster. A small cluster of tissue‐resident memory (TRM) cells was identified based on the expression of canonical TRM markers such as *ZNF683* (encodes for HOBIT), *PRDM1* (encodes for BLIMP1), *ITGA1* (encodes for CD49A), *ITGAE* (encodes for CD103), and *CXCR6* (Figure 1F and Figure S1B). A small population of proliferating cells was also identified by their enrichment for proliferation and cell cycle genes, notably *MKI67* (encodes for Ki‐67) (Figure 1F).

### 2.2 HNSCC TME is Populated with Unconventional CD8^+^ T‐cells

We also identified three clusters of unconventional T‐cells (Figure 1G and Figure S1C). Two of these had gene expression patterns indicative of gamma delta (ɣδ) T‐cell subsets. The third cluster expressed markers corresponding with a mucosal‐associated invariant T (MAIT) cell population. ɣδ T‐cell clusters could be differentiated based on the expression of TCR genes (Figure S1D), marking the two clusters as the Vɣ9Vδ2 T‐cells (G9D2) and non‐G9D2 populations. All unconventional T‐cell populations expressed high levels of *CD3* and *CD8* as previously described [22, 23] (Figure S1E). Differential gene expression revealed that the G9D2 population expressed cytotoxicity markers such as *GZMA, GZMB, GZMH, GNLY, PRF1*, and *NKG7* (Figure S1B,F). Non‐G9D2 γδ T‐cells expressed markers such as *TCF7*, *CD27*, *KLRD1*, and *SELL*. Analysis of differentially expressed transcription factors revealed that these three cell clusters had distinct and unique transcriptional regulatory programs (Figure S1G). For example, G9D2 cells revealed specific enrichment for transcription factors *EOMES*, *ZEB2*, and *ZNF683* (encodes for HOBIT), while non‐G9D2 cells were enriched for *ID3*, *IKZF2*, *TCF7*, and *BACH2*. Meanwhile, MAIT cells demonstrated a distinct pattern of enrichment for transcription factors associated with the MAIT lineage, such as *RORA*, and *ZBTB16* (encodes for PLZF). Altogether, the unconventional T‐cells, TRMs, and proliferative cells, cumulatively represented about ∼10% of TILs within the dataset (Figure 1H).

### 2.3 Ex Vivo TCR Stimulation Leads to the Emergence of Two Transcriptionally Distinct T‐cell Clusters

**FIGURE 2:**
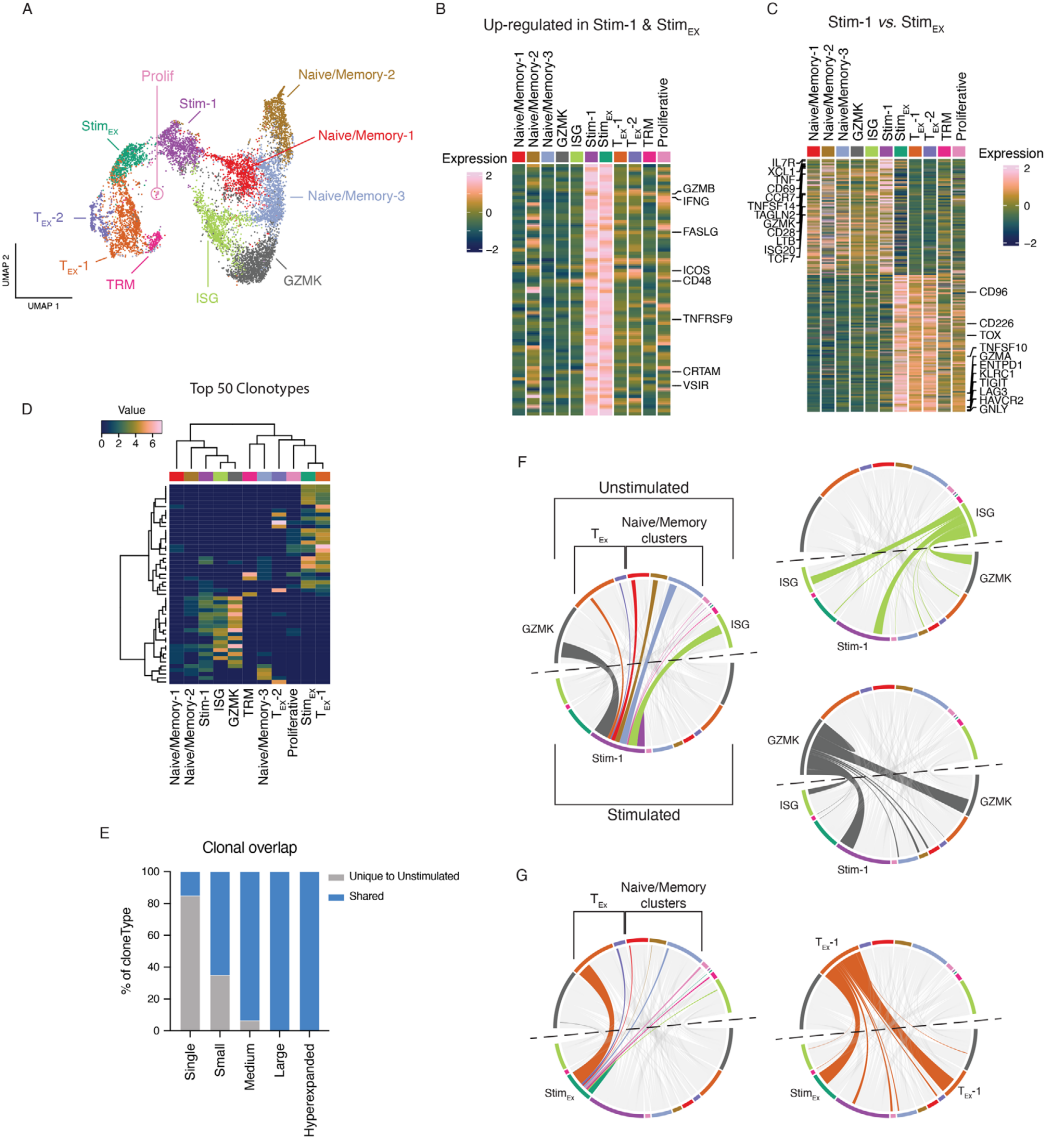
Ex vivo TCR stimulation‐induced transcriptional states develop from distinct unstimulated origins. (A) UMAP projection of CD8^+^ TILs identified in HNSCC patients after removal of unconventional T‐cell subsets. (B) Heatmap of DEGs found to be upregulated (>0.5 log2FC) in both stimulated‐1 and stim‐exhausted clusters, selected genes are annotated. (C) Heatmap of genes found to be significantly differentially expressed (>0.5 log2FC) between stim‐1 and stim‐exhausted clusters, selected genes are annotated. (D) Heatmap of the top 50 most abundant clonotypes found in CD8^+^ HNSCC TILs (ward.D2 clustering and binary distance function). (E) Stacked barplot showing the frequency of each clone size definition that is only found in the unstimulated sample (Unique to Unstimulated) or was also recovered poststimulation (shared). Single (*x* = 1), small (1 < *x* < = 5), medium (5 < *x* ≤ 10), large (10 < *x* ≤ 20) and hyperexpanded (20 < *x* ≤ 150). Where *x* = number of cells with exact CDR3 amino acid sequence. (F) Circos plots depicting the clonal overlap between clusters pre‐ (unstimulated; top arc) and poststimulation (stimulated; bottom arc). Ribbons are colored based on their unstimulated origin. Left column shows ribbons that connect to Stim‐1 cluster whereas right column highlights ribbons that originate from ISG (top) or GZMK (bottom) clusters. (G) Same as (F) with left plot highlighted to show ribbons connecting with Stim‐exhausted (Stim_Ex_) and ribbons in right plot highlighting those that originate from unstimulated T_Ex_‐1 cluster. Data representative of eight patients acquired in one sequencing experiment.

For further analysis, we removed the three unconventional T‐cell clusters from the dataset and recalculated the UMAP coordinates (Figure 2A). We next sought to investigate the two cell clusters that predominantly arose from TCR‐based stimulation. Importantly, both stimulation‐induced clusters shared expression of a number of genes expected following TCR activation, including critical effector molecules such as *IFNG, GZMB*, or *FASLG*, as well as activation markers such as *ICOS* and *TNFRSF9* (encodes for 4‐1BB) (Figure 2B and Tables S1 and S2). However, despite an overlap of activation‐induced transcription, both stimulation‐induced clusters showed distinct patterns of gene expression reminiscent of their origin (Figure 2C and Table S3). For example, the Stim‐1 cluster was enriched for genes such as *IL7R*, *XCL1*, *CD69*, *TNFSF14* (encodes for LIGHT), *CD28*, and *LTB*, whereas the Stim_EX_ cluster expressed high levels of exhaustion markers such as *TOX*, *LAG3*, *HAVCR2* (encodes for TIM‐3) and *CD96*. These basal gene expression profiles seem to overlap with gene expression of other clusters of the dataset. For example, genes enriched in the Stim‐1 cluster were also highly abundant in Naïve/memory, GZMK, and ISG clusters, while genes expressed within the Stim_EX_ cluster were found enriched within the remaining two T_EX_ clusters and to a lesser extent within the TRM and proliferating cell clusters. This overlap suggested the two stimulation‐induced clusters may have arisen from different transcriptional states. To test this hypothesis, we used the single‐cell TCR sequencing data to trace clonal populations between unstimulated and stimulated datasets.

To assess the TCR landscape of HNSCC TILs, we analyzed the top 50 clonotypes detected within the dataset (Figure 2D). This analysis revealed clonotypes observed in the Stim‐1 cluster were also found within ISG and GZMK clusters. In contrast, the Stim_EX_ cluster shared many highly abundant clones with the T_EX_‐1 cluster, indicating clonal overlap between these populations. To explore this further, we next traced clones pre‐ or poststimulation to investigate the clonal overlap with respect to stimulation and cluster identity. However, this analysis relied on the assumption that clones were sufficiently represented in both pre‐ and poststimulation datasets. Indeed, it was observed that when clones are represented in two or more T‐cells (clone size small), >60% of clones are captured within the stimulated dataset (i.e., shared) (Figure 2E). Therefore, we proceeded with tracing the transcriptional responses of shared T‐cell clones by linking their cluster identity pre‐ and poststimulation. We observed that cells from the Stim‐1 cluster largely overlapped with unstimulated ISG and GZMK clusters (Figure 2F). Tracing unstimulated ISG clones, we observed clonal overlap that suggested stimulated ISG cells, either maintain their identity or adopt a GZMK or Stim‐1 transcriptional phenotype. Similarly, unstimulated GZMK cells either retained GZMK identity or adopted ISG or Stim‐1 transcriptional profiles poststimulation. In contrast, clones from the Stim_EX_ cluster were predominantly found to overlap with the unstimulated T_EX_‐1 cluster with a minimal contribution from other unstimulated clusters (Figure 2G). As predicted, unstimulated T_EX_‐1 cluster clones overlapped with stimulated Stim_EX_ or T_EX_‐1 clusters. Interestingly, this analysis also revealed that TCR stimulation was capable of inducing a gene signature associated with T‐cell activation in a subset of transcriptionally terminally exhausted T‐cells (TCF7‐TOX+PD1+) (Figures 1E and 2B,C)

### 2.4 ISG Cells Largely Retain Their Transcriptional Identity Upon TCR Stimulation

**FIGURE 3:**
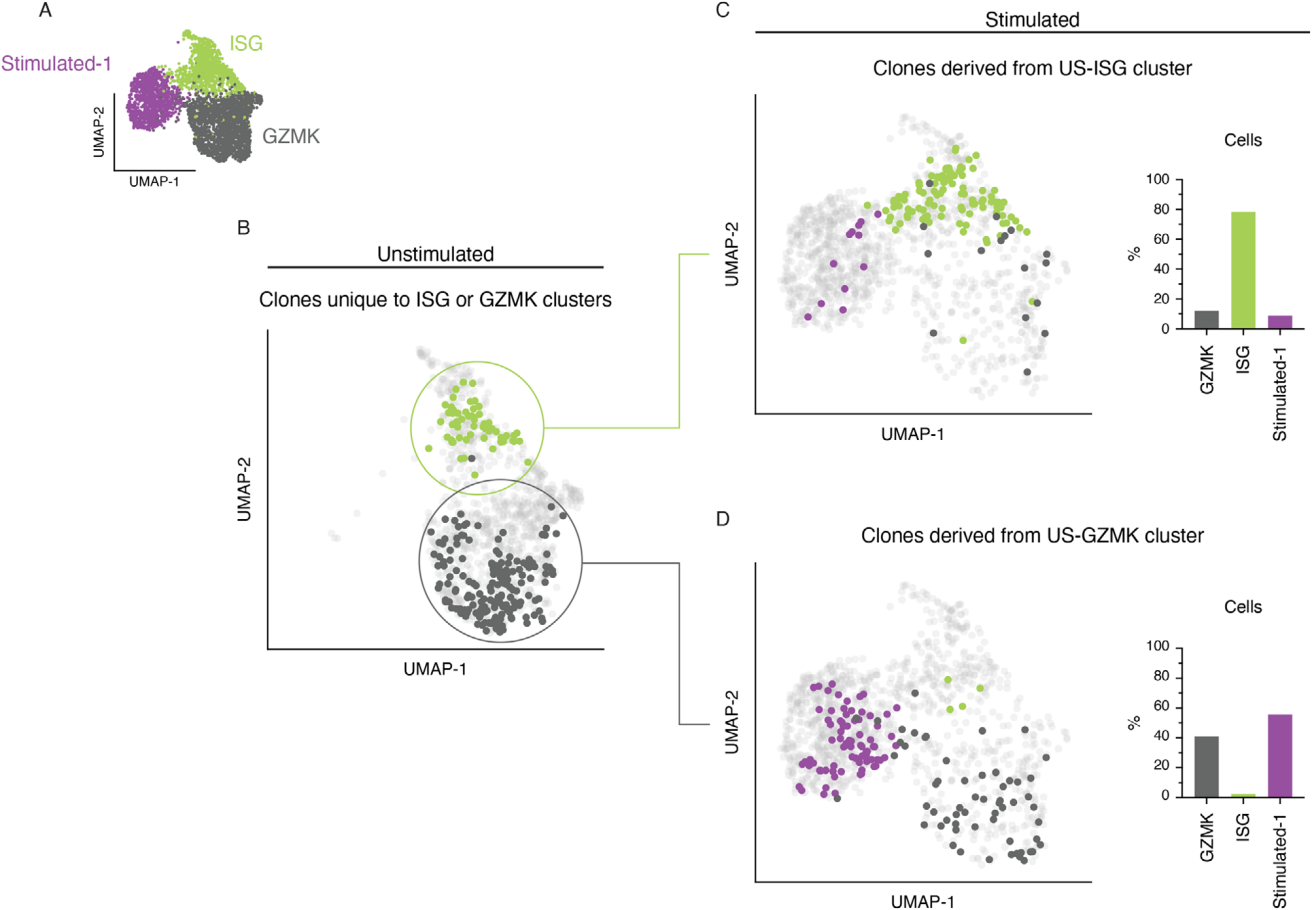
ISG cells are poorly transcriptionally responsive to TCR stimulation. (A) UMAP projection of Stimulated‐1, ISG, and GZMK clusters both from unstimulated and stimulated datasets. (B) UMAP projection highlighting TCR clones uniquely found within unstimulated ISG cluster (green) or unstimulated GZMK cluster (black). (C) UMAP projection and quantification highlighting the distribution of unique US‐ISG clones poststimulation. Barplots quantify the frequency of cells poststimulation. (D) same as (C) but for US‐GZMK clones poststimulation. Data representative of eight patients acquired in one sequencing experiment.

Given the clonal overlap between ISG, GZMK, and Stim‐1 clusters, we next sought to investigate their responsiveness to TCR stimulation. To this end, we first isolated these clusters and projected the cells onto their own UMAP coordinates (Figure 3A). Subsequently, we identified clones that were present in both pre‐ and poststimulation datasets. However, cells from any particular clonotype may be distributed across numerous clusters prestimulation. Therefore, the poststimulation transcriptional phenotype may result from the stimulation of cells from any prestimulation cluster. To mitigate this confounding factor, we further filtered for clones for which their constitute cells were entirely contained within the ISG or GZMK clusters in the unstimulated dataset. Thus, any poststimulation transcriptional phenotype could be better ascribed to the stimulation of cells with an ISG or GZMK transcriptional identity. This filtering resulted in the retention of 26 and 53 unique clonotypes within unstimulated ISG or unstimulated GZMK clusters, respectively (Figure 3B). Following TCR stimulation, the majority of ISG T‐cells retained their transcriptional identity (Figure 3C). In contrast, over 50% of unstimulated GZMK T‐cells adopted a Stim‐1 transcriptional identity following stimulation (Figure 3D), while the remaining proportion retained their GZMK identity. Interestingly, there was minimal adoption of an ISG signature following stimulation of GZMK clones.

### 2.5 A Type I Interferon Signature is Associated with Reduced Transcriptional Activity in ISG TILs

**FIGURE 4:**
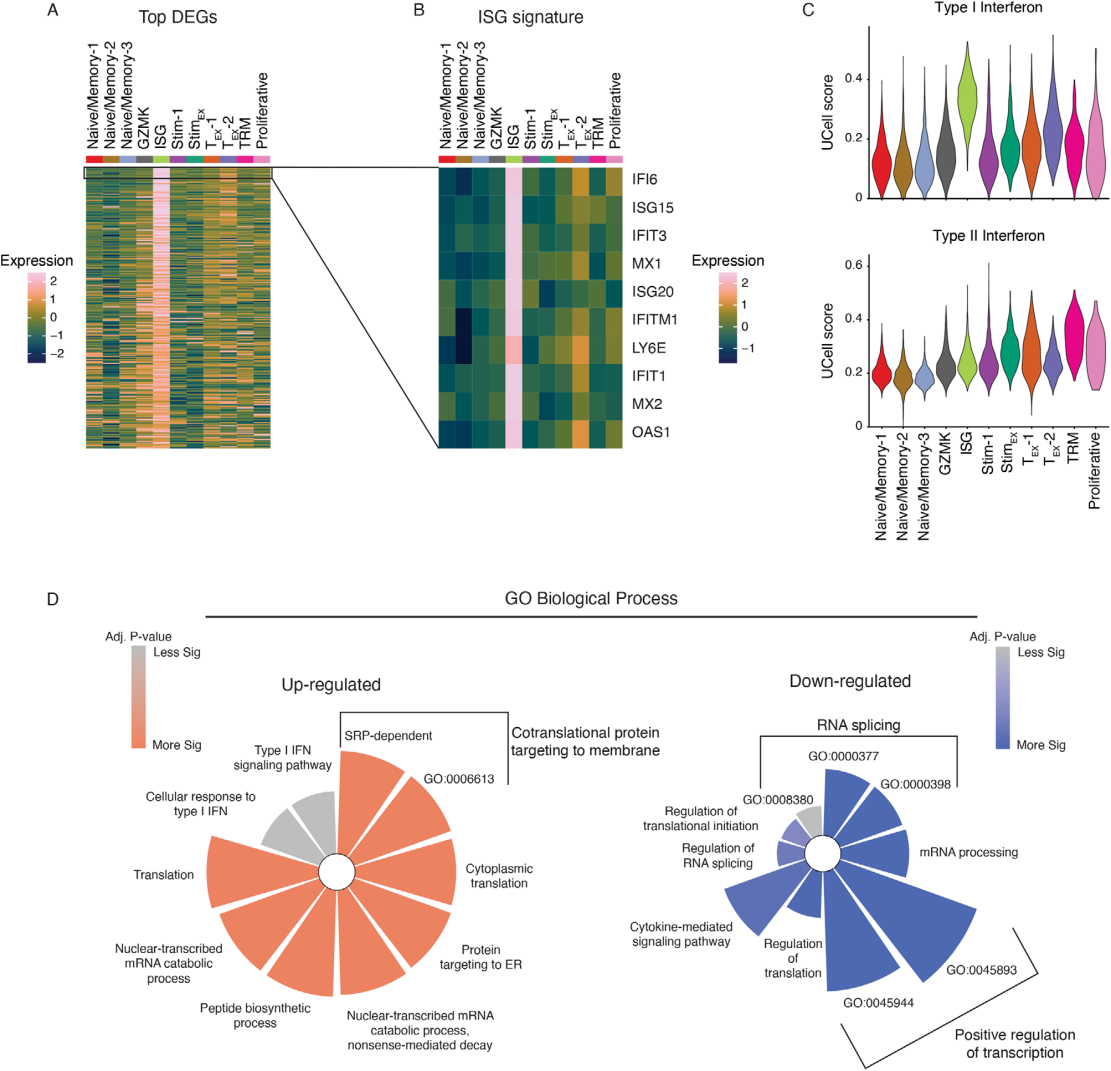
ISG cells are enriched for a type I interferon signature and are associated with reduced transcriptional. (A) Heatmap showing the top upregulated DEGs (> 0.25 Log2FC) identified in ISG cluster. (B) Heatmap showing top 10 DEGs identified in ISG cluster. (C) Violin plots of UCell scores for a type I interferon (top) or a type II interferon (bottom) gene signatures. (D) Gene ontology analysis for the top upregulated (left) and downregulated (right) biological processes identified in the ISG cluster. Data representative of eight patients acquired in one sequencing experiment.

Given the diverse role of interferon signaling for the function of tumor‐infiltrating T‐cells, the relevance of ISG cells during tumor progression and immunotherapy remains elusive. We performed differential gene expression analysis and revealed a dominant signature enriched within the ISG population (Figure 4A and Table S4). The top 10 differentially expressed genes identified within the ISG cluster were almost all found downstream of interferon signaling (Figure 4B). To understand the type of interferon signaling responsible, clusters were scored for genes contributing to a type I or type II interferon response (Figure 4C). Results showed the ISG cluster had enrichment for a type I, but not a type II interferon gene signature. Gene Ontology (GO) analysis was performed on the differentially up‐ or downregulated genes within the ISG cluster relative to other clusters to unravel dominant biological processes associated with ISG cells. This analysis revealed a broad increase in translation‐related terms and type I IFN signaling responses (Figure 4D). Interestingly, downregulated genes were enriched for GO terms associated with transcriptional regulation. This finding could explain our previous observation, that ISG cells poorly adopt new transcriptional states following TCR stimulation.

### 2.6 ISG Cells are Enriched in CD8^+^ TILs Across Various Tumor Types

To establish whether ISG cells could be identified in other microenvironments, we generated a specific gene signature using the top 10 differentially expressed genes from ISG cells within our data set (Figure 4B). We next examined if this signature could identify ISG cells in a publicly available HNSCC dataset in which an ISG cluster had previously been reported [24]. Indeed, using our curated ISG signature, we were able to correctly identify a cluster of cells enriched for type I interferon genes (Figure S2A).

**FIGURE 5:**
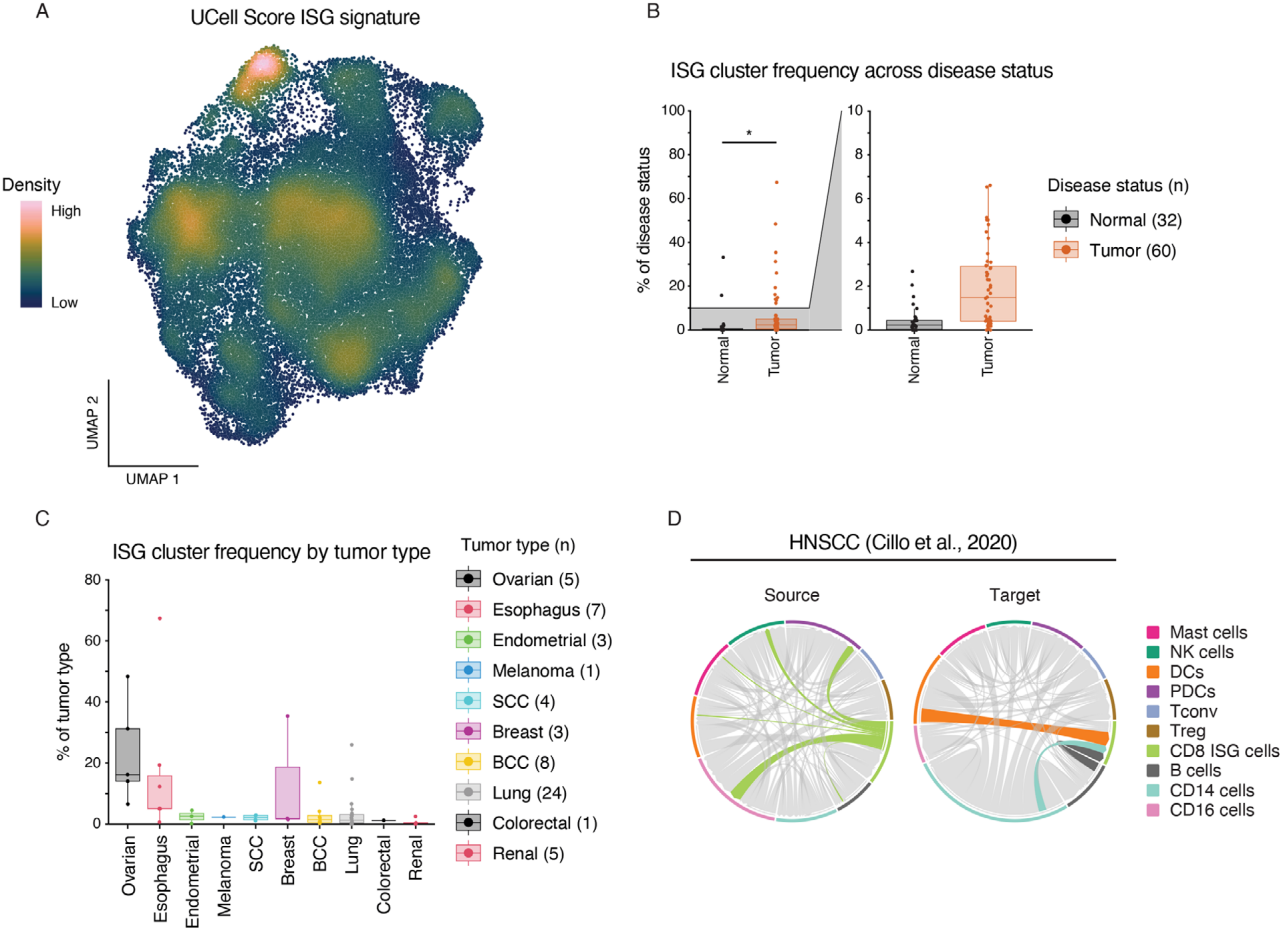
Cells with a type I interferon signature can be found across various tumor entities and are enriched within tumor tissue. (A) UMAP coordinates of CD8^+^ T‐cells in a pan‐cancer dataset overlaid with UCell score for ISG signature. Data representative of 67 patients. (B) Boxplot showing ISG cluster frequency per donor across normal and tumor tissue samples. (C) Boxplot showing ISG cluster frequency within tumor samples per donor across tumor types within the dataset. (D) Circos plots generated using the top 20 interactions for each source (left) or target (right) with ribbons highlighting interactions originating from ISG cluster (left) or terminating in ISG cluster (right), ribbons colored by source. Data representative of 26 patient samples. *p*‐value calculated using a two‐tailed *t*‐test. (*n*) value indicates the number of unique donors. ns = *p* > 0.05, *p < 0.05, **p < 0.01, ***p < 0.001, ****p < 0.0001.

To better understand the abundance of ISG cells within CD8^+^ T‐cells in healthy and malignant tissues, we scored cells from a pan‐cancer dataset for our ISG signature [25]. Indeed, we could identify a fraction of T‐cells highly enriched for our ISG‐signature (Figure 5A). Next, we assessed the frequencies of ISG cells across normal and tumor tissues. Here, we found ISG cells to be significantly increased in tumor tissues, relative to normal tissue (Figure 5B). ISG cells were most frequent in Ovarian and Esophageal tumor types but also detected to various degrees among all other tumor types assessed (Figure 5C). As expected ISG cells were solely enriched for type I but not type II IFN genes (Figure S2B). We also assessed a COVID‐19 dataset including some Influenza samples to determine if ISG cells are also enriched in the blood of virally infected patients [26]. Indeed, in both conditions we observed a population of CD8^+^ T‐cells enriched for our ISG signature (Figure S2C) with a higher frequency in disease compared with healthy control samples (Figure S2D), suggesting that the ISG cluster phenotype is not restricted to tumor immunity.

Finally, to better understand the functional role of ISG cells within the TME, we employed cell‐cell communication analysis. Utilizing a published HNSCC dataset containing an array of immune cell subsets from both HPV^+^ and HPV^−^ patients [24], we revealed that ISG cells served as the source for interactions with CD16 positive cells, as well as with NK cells and plasmacytoid dendritic cells (PDCs) (Figure 5D). ISG cells were also found to be a target for DC, B cell, and CD14 cell interactions. Hence, this data suggests ISG cells interact with key innate immune cell subsets within the TME and thus potentially are important orchestrators of antitumor immunity.

## 3 Discussion

HNSCC is a prevalent and complex disease with numerous etiological influences. For example, viral co‐infection with HPV in Oropharyngeal HNSCC is associated with a better prognosis, especially in early‐stage disease. As such, HPV‐negative HNSCC presents as a more therapeutically challenging entity. Therefore, we sought to expand the knowledge base of CD8^+^ tumor‐infiltrating lymphocytes (TILs) landscape, specifically in treatment‐naïve HPV‐negative HNSCC patients. We employed a multimodal sequencing approach, together with an ex vivo TCR stimulation, to facilitate tracing of transcriptional profiles and response capacity in CD8^+^ T‐cell subsets.

Single‐cell RNAseq of immune cell subsets has enabled in‐depth mapping of the cellular heterogeneity of various disease conditions. However, traditionally this methodology only assesses the transcriptional state of cells *ex vivo*. Thus, capturing a snapshot of the cellular transcriptomic landscape. However, by leveraging an ex vivo perturbation coupled with sequencing approaches, others have ascertained both ex vivo profiles and their subsequent activation potentials. For example, a study by [27] performed *ex vivo* TCR stimulation on T‐cells isolated from several healthy donor tissues. The authors were able to define both conserved tissue signatures as well as the activation states of T‐cells [27]. Using a similar approach, we included TCR sequencing to facilitate the tracing of transcriptional responses within clonal populations of tumor‐infiltrating T‐cells. Notably, we observed two unique T‐cell clusters specifically induced by TCR stimulation. Transcriptional signatures and clonal overlap suggest these populations arose via stimulation of distinct *ex vivo* subsets. Importantly, we observed cells that displayed a transcriptional program of terminal exhaustion (*TCF7*‐*TOX*+*PDCD1*+*TIM3*+). However, we observed that those populations retained substantial capacity to respond to TCR stimulation, at least in vitro, when alleviated of their tumor microenvironmental milieu [28]. These data posit that transcriptionally exhausted cells may retain the substantive capacity to respond to stimulation. Indeed, numerous scRNAseq studies have identified clusters of exhausted cells that simultaneously express high levels of effector molecules [29, 30]. However, since most scRNAseq studies do not incorporate a stimulation step, the response capability of transcriptionally exhausted cells has been underappreciated. Resistance to PD‐1 therapy is still a frequently observed clinical scenario in HNSCC and the presence of terminally exhausted T‐cells has been proposed to be a possible cause of resistance toward PD‐1 therapy [31]. Our data tentatively suggests that combination strategies to break down tumor microenvironmental signals might be a strategy to overcome PD‐1 resistance, indeed re‐invigoration of exhausted T‐cells is an active area of investigation [32]. Nonetheless, these observations highlight the need for multimodal data approaches to identify prototypic exhausted T‐cells while urging caution against defining exhaustion solely based on transcriptional profiles.

IFN‐I signaling in CD8^+^ T‐cells is associated with both anti‐ and protumoral function [18]. Therefore, the clinical implications of an ISG‐rich population are poorly understood. Substantial challenges impede the experimental investigation of these cells and as such our multimodal sequencing approach has provided a comprehensive investigation of this population. Our analysis has revealed that CD8+ ISG cells are a common feature of solid malignancies and are specifically enriched within tumor tissue. Furthermore, we have found that ISG cells are clonally related to GZMK‐expressing CD8^+^ TILs. However, experimental perturbation revealed that ISG cells are transcriptionally stable and inert to TCR stimulation. However, it remains to be determined whether ISG cells are entirely unresponsive or if they simply possess an altered threshold for activation; potentially requiring more prolonged or intense stimuli to trigger transcriptional changes. Although, numerous unknowns remain and ultimately further experimentation is required to understand the functional implications of this differentiation pathway and these cellular states.

This is not the first report to describe a population of cells enriched with interferon‐stimulated genes. Indeed, numerous others have observed similar populations amongst malignant, infectious, and healthy tissues [24, 30, 33, 34]. However, the absence of specific cell‐surface markers has hindered investigation efforts. Thus far, reports of this population have been limited to mere observation of their appearance. Illustrative of this, Wang et al. [34] identified a subset of ISG cells within sequencing data of healthy PBMCs. Despite their efforts, the authors were unable to experimentally isolate this population and thus were limited in the functional analysis that could be performed. Therefore, alternative markers and/or strategies to identify and isolate cells with this cellular state are required. In the absence of this, our multimodal sequencing and experimental perturbation approach has provided novel insights into ISG CD8^+^ TILs.

The relationship between GZMK and ISG cells is notable as others have demonstrated that GZMK expression within solid tumors is associated with improved patient outcomes [35, 36]). However, the nature of this association is unclear, as GZMK is usually correlated with innate cells and naïve phenotypes. For example, GZMK is more dominantly expressed within immature NK cells. However, GZMK expression within CD8+ T‐cells is predominantly observed within central memory and effector memory subsets [37]. Thus, supporting the notion that GZMK expression within CD8^+^ T‐cells may correlate with a favorable prognosis. Although, it has been observed that GZMK^+^ CD8^+^ T‐cells are poorly cytotoxic and instead produce IFNγ [37, 38]. Interestingly, others have reported differential effects of TCR or cytokine stimulation on GZMK expression. Namely, that TCR stimulation induces the release of GZMK and an increase in GZMB expression. Conversely, cytokine‐based stimulation drives the accumulation of GZMK [37]. These findings are consistent with our results which demonstrated that TCR‐based stimulation drives GZMK cells to down‐regulate GZMK and upregulate GZMB as they differentiate toward a more terminal effector phenotype. Therefore, these data suggest GZMK positivity marks CD8^+^ T‐cells which are not yet terminally differentiated and instead possess a more memory‐like phenotype. Given the above model, the accumulation of ISG cells could prevent the development of more terminally differentiated antitumoral responses via GZMK intermediaries. However, GZMK^+^ CD8^+^ T‐cells have been observed within tumor stroma and have been implicated in poor prognosis [39]. Additionally, GZMK CD8^+^ TILs have been described as a transition state on the trajectory toward exhaustion [36, 40]. This is consistent with reports showing IFN‐I signaling as a driver of T‐cell exhaustion [19, 20]. Therefore, the functional consequences of ISG and GZMK TILs are poorly defined. Further studies are required to better understand the dynamics and function of T‐cell clusters infiltrating tumor tissues.

This study sheds light on the complex landscape of CD8^+^ TILs in the context of HPV‐negative HNSCC. Through a multimodal sequencing approach and *ex vivo* perturbation, we identified a previously overlooked population of CD8^+^ TILs enriched for ISGs. These ISG cells exhibited transcriptional inertness to TCR‐based stimulation and were found to be clonally related to granzyme K‐expressing cells. Furthermore, ISG cells were found to be specifically enriched within tumor tissue and could be identified in various tumor entities. The specific enrichment of ISG cells within tumor tissue and their unresponsive nature suggest the ISG cells may represent an undesirable differentiation path. Therefore, ISG‐cells may impede the development of an effective antitumoral T‐cell response. However, further investigation is warranted to delineate the functional implications of ISG cells and to explore potential therapeutic avenues. Our findings contribute to a deeper understanding of the immune landscape in HPV‐negative HNSCC and highlight an overlooked T‐cell phenotype.

## 4 Materials and Methods

### 4.1 Patient Samples

A total of eight patients who had provided informed consent were included in this study. Samples were obtained from surgical resections of primary HNSCC tumors. All patients presented with oral cavity squamous cell carcinoma and were confirmed to be human papillomavirus (HPV) negative. Fresh HNSCC tumors were collected at the time of resection of the primary tumor and sampled by a pathologist prior to fixation. Fresh tissue was processed to isolate tumor cells and immune cells prior to preservation and storage in liquid nitrogen. The patients enrolled in this study were treatment naïve and characteristics can be found in Figure 1A.

### 4.2 Single‐cell RNA Sequencing

Cells from each patient were cultured as single‐cell suspensions and were either stimulated using CD3/CD28 beads or left unstimulated for a duration of 5 h. Following culture, the cells were sorted using fluorescence‐activated cell sorting to isolate live CD45^+^CD3^+^CD4^−^CD8^+^ cells. Patient samples were sequenced as two unstimulated and two stimulated samples where each sequencing sample represented a pool of four patients. As such, approximately 10,000 cells per sample pool were carried forward into the 10x Genomics Single‐cell 5’ library pipeline. The libraries were sequenced using a NextSeq 550 (Illumina). The sequencing was performed at QIMR Berghofer Medical Institute.

### 4.3 scRNAseq Preprocessing

Sequencing reads were processed using cellranger (version 3.1.0) and reads were aligned to the human reference genome GRCH38‐3.0.0 [41]. Output from cellranger was processed using Seurat (version 4.3.0) with additional functionality provided by SeuratDisk (version 0.0.0.9020) and SeuratObject (version 4.1.3) [42, 43, 44]. Each sequencing sample was filtered to keep only cells that had a minimum of 200 features and keep features that were detected in a minimum of three cells. Subsequently, the two unstimulated samples were merged and the two stimulated samples were merged to give two Seurat objects. These Seurat objects were further filtered to remove cells with greater than 2500 features or greater than 10% mitochondrial content. Filtering resulted in 5785 cells with 15,429 features in the unstimulated dataset and 6042 cells with 15,618 features in the stimulated dataset. Datasets were normalized using LogNormalisation with a scale factor of 10,000. Subsequently, mitochondrial percentage and nCount variables were regressed using a linear model. Unstimulated and Stimulated datasets were integrated using the Seurat integration pipeline. Unless otherwise stated integration functions/pipeline was executed using default function variables. Integration anchors were calculated using “cca” reduction, “LogNormalize” as a normalization method, and “rann” as the Nearest Neighbour method. Integration resulted in a dataset of 18,295 features across 11,827 cells.

### 4.4 scRNAseq Analysis

#### 4.4.1 Dimension Reduction and Cluster Identification

The top 30 PCAs were calculated on the integrated dataset and nearest‐neighbors were computed using the top 20 dimensions. Clusters were determined using a cluster resolution of “0.4”. UMAP in Figure 1 was generated using top 20 PCA dimensions, the “uwot” algorithm, n.neighbors = 30, and min.dist = 0.3. Following UMAP dimension reduction calculation, clusters were investigated both with manually curated gene signatures and with the use of SingleR (version 2.0.0) to classify cells using data from celldex (version 1.8.0) [45]. Two low abundance clusters were removed that were identified as either having high mitochondrial content or a myeloid signature. UMAP projection was recalculated following the removal of these clusters, using the same parameters as previously stated. Therefore, after cluster identification the dataset contained 20,295 features across 11,658 cells with 5724 cells from the unstimulated treatment condition and 5934 cells from the stimulated treatment condition. Subsequently, unconventional T‐cell clusters were subsetted from the dataset resulting in unconventional T‐cell‐only and CD8‐only datasets. UMAP projections were recalculated for these datasets using the top 20 PCA dimensions, n.neighbors = 50, and a min.dist of 0.1 for the CD8‐only dataset or 0.5 for the unconventional T‐cell‐only dataset. The unconventional T‐cell‐only dataset consisted of 20,295 features across 970 cells. The CD8‐only dataset consisted of 20,295 features across 10,688 cells, 5165 of which originated from the unstimulated treatment condition and 5523 from the stimulated treatment condition.

#### 4.4.2 Differential Gene Expression

Calculations to determine differentially expressed genes between clusters or conditions were performed using the Wilcox test implemented via the standard Seurat analysis pipeline. Analysis was performed using the RNA data slot of the Seurat object.

#### 4.4.3 Differentially Expressed Transcription Factors

To determine the differential expression of transcription factors, the list of differentially expressed genes was cross‐referenced with a curated database of RNA polymerase II regulated transcription factors (TFcheckpoint; http://www.tfcheckpoint.org).

#### 4.4.4 Gene Ontology Analysis

Briefly, differentially expressed genes for the ISG cluster were identified using Seurat's FindMarkers() function. Genes identified as significantly (adjusted *p*.value < 0.5) up‐ or downregulated were then passed to the enrichR package (version 3.1) to identify enriched terms using the GO_Biological_Process_2021 database [46]. The top 10 enriched terms were then visualized using SCpubr (version 1.1.1) [47].

#### 4.4.5 Signature Scoring

Signature score was calculated using UCell (version 2.2.0) [48] with signatures for type I and II IFN obtained from [49].

#### 4.4.6 Cell–Cell Communication Analysis

Cell‐cell communication was performed using the R package “liana” (version 0.1.12) [50]. In brief, cell–cell communication networks were calculated using the following methods “natmi”, “connectome”, “logfc”, “sca”, and “cellphonedb”. The scores from these methods were subsequently aggregated and only interactions concordant between methods were kept. This analysis followed the recommended analytical pipeline for the “liana” package.

### 4.5 scRNAseq Visualization

#### 4.5.1 Imputation

Imputation of gene expression was performed and used in certain visualizations where indicated. Imputed values were not used for any downstream analysis and were exclusively used in indicated visualizations. Imputation was performed using the “RunALRA” function in SeuratWrappers (version 0.3.1) and increased the percentage of non‐zero values in the dataset from 29.63% to 38.95% [51].

#### 4.5.2 Density‐Based UMAP Visualization

The Nebulosa package (version 1.8.0) and scCustomize package (version 1.1.1) were used to visualize gene expression on UMAP projections and expression density [52, 53].

#### 4.5.3 Color Scheme

Where possible the uniform, colorblind‐friendly batlow [54] color pallet was used for data visualization. The color palette was accessed using the Scico package (version 1.3.1) [55].

### 4.6 Single‐Cell TCR Sequencing Analysis

#### 4.6.1 Preprocessing

Single‐cell TCR sequencing data were aligned using cellranger pipeline (version 3.1.0) to the human VDJ reference (vdj_GRCh38_alts_ensembl‐3.1.0‐3.1.0). TCR data was subsequently processed using scRepertoire (version 1.8.0) [56]. TCR data was filtered such that if cells had multiple alpha or beta chains identified, only the top expressing chain was retained. Additionally, unless otherwise stated, clone identity was defined by the CDR3 amino acid sequence.

#### 4.6.2 Clone Size Definitions

Abundance of clones was calculated per stimulation condition and binned according to the following definitions. Single (x = 1), small (1 < *x* < = 5), medium (5 < *x* ≤ 10), large (10 < *x* ≤ 20) and hyperexpanded (20< *x* ≤ 150). Where *x* = number of cells with exact CDR3 amino acid sequence. Size cut‐offs were determined empirically using summary statistics of clone abundances across the dataset.

### 4.7 External Datasets

#### 4.7.1 uTILity

The pan‐cancer “uTILity” dataset was acquired from [25] circa 13.10.2022. The dataset was filtered to retain only cells identified as CD8 T‐cells and only Tumor and Normal tissue types were retained. The subsetted dataset was normalized and reintegrated using the harmony package (version 0.1.1) to remove “Cohort” effect [57]. UMAP coordinates and clusters were recalculated following harmonization, using the standard Seurat analysis pipeline.

#### 4.7.2 HNSCC

For validation of ISG gene signature and cell‐cell communication analysis, the HNSCC TILs dataset published in [24] was used. Processed data were downloaded from (GSE139324) [https://www.ncbi.nlm.nih.gov/geo/query/acc.cgi?acc = GSE139324]. Metadata for this dataset was obtained through contact with the lead author/s.

#### 4.7.3 COMBAT Dataset

The Covid‐19 and Influenza scRNAseq dataset was downloaded from [58] https://zenodo.org/records/6120249.

### 4.8 Figure Preparation

Figures were arranged and formatted using Adobe Illustrator (version 27.5) and/or GraphPad Prism (version 9).

## Author Contributions

Dillon Corvino: Conceptualization, methodology, software, formal analysis, investigation, data curation, writing (original draft), visualization, and supervision. Martin Batstone, Brett G. M Hughes, Sally Pearson, Jason Madore, Kevin Thurley, Michael Hölzel, Nicholas Borcherding, and Nicholas Borcherding: Resources. Tim Kempchen, Lambross T. Koufariotis, and Dilan Pathirana: Software, formal analysis. Susanna S Ng: Conceptualization and investigation. Nazhifah Salim, Franziska Schneppenheim, Denise Rommel, and Ananthi Kumar: Investigation. Lambross T. Koufariotis: Data curation. Nicholas Borcherding: Software, formal analysis, data curation, and visualization. Martin Batstone: Conceptualization, methodology, writing (original draft), and supervision. Tobias Bald: Conceptualization, investigation, writing (original draft), supervision, funding acquisition, and project administration. All authors read and approved the final manuscript.

## Ethics Statement

Ethical approval for this study was obtained from the Royal Brisbane and Women's Hospital Human Research Ethics Committee and the QIMR Berghofer Human Research Ethics Committee, HREC/18/QRBW/245.

## Conflicts of Interest

N.B. is a current employee of Omniscope, Inc. and has consulted for Starling Biosciences and Santa Ana Bio in the last 36 months. All other authors declare no conflicts of interest.

### Peer review

The peer review history for this article is available at https://publons.com/publon/10.1002/eji.202451371

## Supporting information




**Supplementary Figure 1: Transcriptional profile of CD8+ T‐cell and unconventional T‐cell subsets in head and neck squamous cell carcinoma**. (A) Stacked barplot showing the relative proportion of each cluster by stimulation status. (B) Stacked violin plots of key genes across identified clusters. (C) UMAP projection of unconventional T‐cells identified within sequencing dataset. (D) Heatmap of unconventional T‐cell clusters showing gamma‐delta TCR genes detected and key markers of MAIT‐cells. (E) Stacked violin plots of key T‐cell receptor genes. (F) Heatmap of top differentially expressed genes (log2FC > 1) with selected genes annotated. (G) Heatmap of the average expression of differentially expressed transcription factors. Data representative of eight patients acquired in one sequencing experiment.

**Supplementary Figure 2: Type I interferon‐stimulated cells are present in patients with viral infection**. (A) Violin plot of UCell score for ISG signature across the CD8+ T‐cell clusters within Cillo et al., 2020 dataset. Data representative of 26 patients. (B) Violin plots of UCell score for type I interferon (left) or type II interferon (right) gene signatures across indicated entities grouped by cells from identified ISG cluster or all remaining cell clusters. Data representative of 5 Ovarian or 7 Esophageal patients. (C) UMAP projection of CD8+ T‐cells from the COVID‐19 Multi‐omics Blood Atlas Consortium showing the density of UCell score for ISG signature. Data representative of 80 samples. (D) Boxplot showing frequency of ISG cluster by disease type per donor. (n) value indicates the number of unique donors. *p*‐value calculated using a two‐tailed *t*‐test. ns = *p* > 0.05, *p < 0.05, **p < 0.01, ***p < 0.001, ****p < 0.0001

**Supplementary Table 1: Differentially expressed genes between clusters**. This table presents the results of a Wilcoxon Rank Sum test to identify differentially expressed genes between clusters in the dataset. This analysis was performed using the “FindAllMarkers” function from the “Seurat” package, with a log fold threshold of 0.25 and the “only.pos” parameter set to “FALSE”. All other parameters were left at their default values.

**Supplementary Table 2: Differentially expressed genes upregulated in Stim‐1 and Stim‐exhausted clusters** This table presents the results of a Wilcoxon Rank Sum test to identify differentially expressed genes between clusters in the dataset. This analysis was performed using the “FindAllMarkers” function from the “Seurat” package, with a log fold threshold of 0.25 and the “only.pos” parameter set to “FALSE”. All other parameters were left at their default values. The results were then filtered to include only significant genes (adjusted *p*‐value < 0.05) found in Stim‐1 or Stim‐exhausted clusters. Additionally, genes were further filtered to retain those that were upregulated (average log2 fold change > 0.5) and significantly differentially expressed in both Stim‐1 and Stim‐exhausted clusters.

**Supplementary Table 3: Differentially expressed genes in Stim‐1 vs. Stim‐exhausted clusters** This table presents the results of a Wilcoxon rank sum test to identify differentially expressed genes between the Stim‐1 and Stim‐exhausted clusters. This analysis was performed using the “FindMarkers” function from the “Seurat” package, with a log fold threshold of 0.25 and the “only.pos” parameter set to “FALSE”. All other parameters were left at their default values.

**Supplementary Table 4: Differentially expressed genes upregulated in ISG cluster** This table presents the results of a Wilcoxon Rank Sum test to identify differentially expressed genes within ISG relative to all remaining clusters. This analysis was performed using the “FindMarkers” function from the “Seurat” package, with no log fold threshold or minimum percentage. All other parameters were left at their default values. The results were then filtered to include only significantly (adjusted *p*‐value < 0.05) upregulated (average log2 fold change > 0.5) genes.

## Data Availability

The data that support the findings of this study are available on request from the corresponding author. The data are not publicly available due to privacy or ethical restrictions. All code used to generate figures can be found under the relevant repository at https://github.com/BaldLab.
