# Supplementary figures and images for: Type I Interferon Drives a Cellular State Inert to TCR‐Stimulation and Could Impede Effective T‐Cell Differentiation in Cancer
Source: Eur J Immunol. 2024 Nov 12;55(1):e202451371. doi: 10.1002/eji.202451371 (PMC11739669; doi:10.1002/eji.202451371)

**A**

Cluster contribution by stimulation status

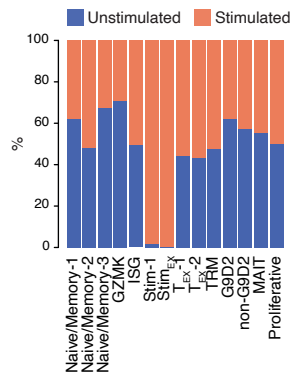

G9D2

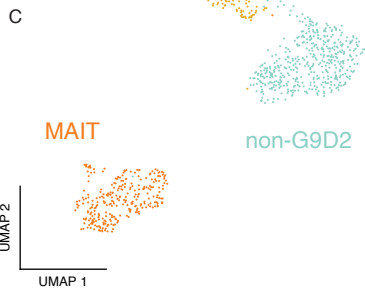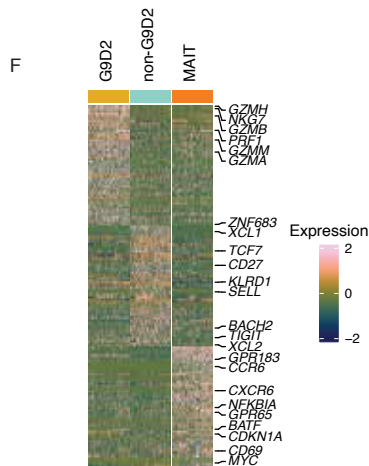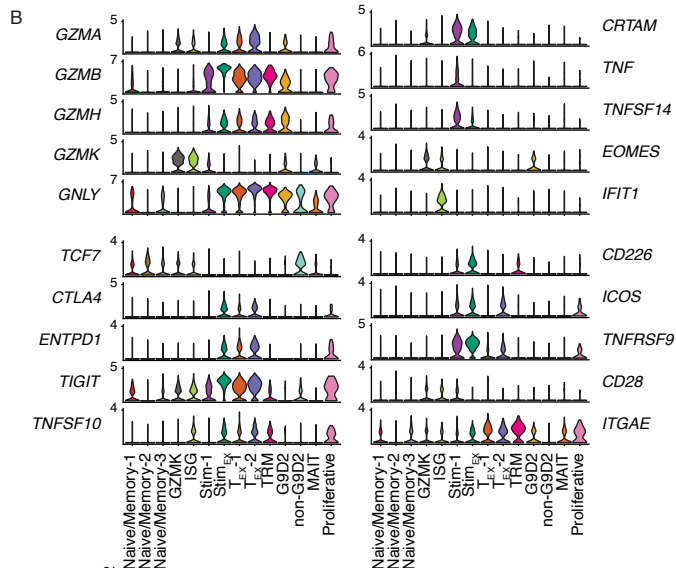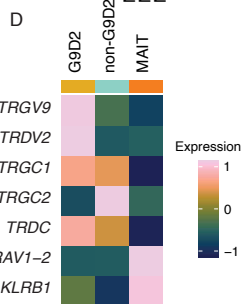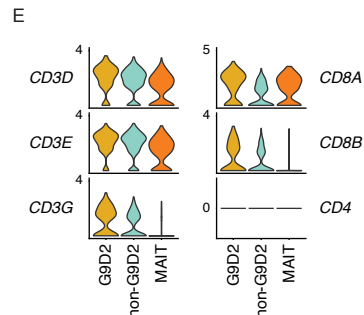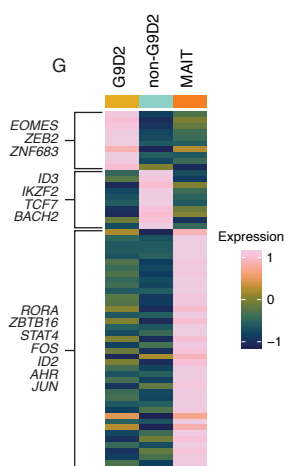

Supplement: Supplementary file 1 — Supplementary Figure 1: Transcriptional profile of CD8+ T‐cell and unconventional T‐cell subsets in head and neck squamous cell carcinoma. (A) Stacked barplot showing the relative proportion of each cluster by stimulation status. (B) Stacked violin plots of key genes across identified clusters. (C) UMAP projection of unconventional T‐cells identified within sequencing dataset. (D) Heatmap of unconventional T‐cell clusters showing gamma‐delta TCR genes detected and key markers of MAIT‐cells. (E) Stacked violin plots of key T‐cell receptor genes. (F) Heatmap of top differentially expressed genes (log2FC > 1) with selected genes annotated. (G) Heatmap of the average expression of differentially expressed transcription factors. Data representative of eight patients acquired in one sequencing experiment. [file EJI-55-e202451371-s006.pdf]

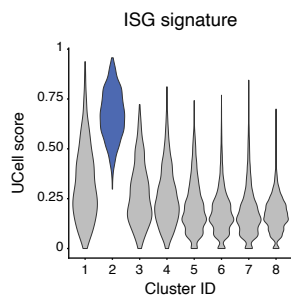

B

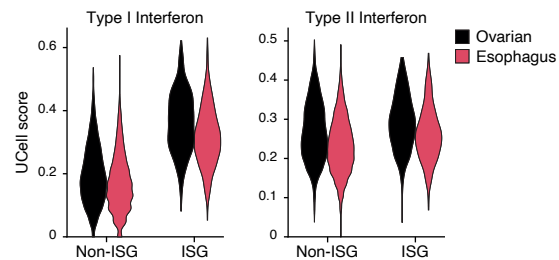

COvid-19 Multi-omics Blood ATlas (COMBAT) Consortium

C

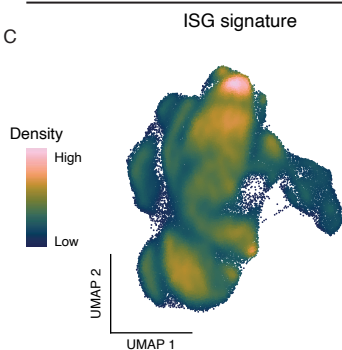

D

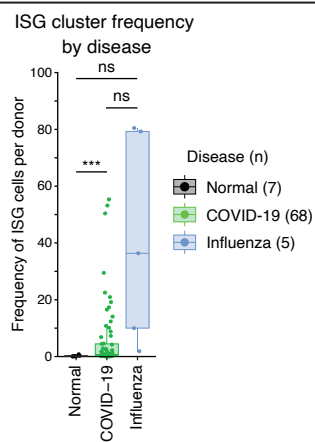

Supplement: Supplementary file 2 — Supplementary Figure 2: Type I interferon‐stimulated cells are present in patients with viral infection. (A) Violin plot of UCell score for ISG signature across the CD8+ T‐cell clusters within Cillo et al., 2020 dataset. Data representative of 26 patients. (B) Violin plots of UCell score for type I interferon (left) or type II interferon (right) gene signatures across indicated entities grouped by cells from identified ISG cluster or all remaining cell clusters. Data representative of 5 Ovarian or 7 Esophageal patients. (C) UMAP projection of CD8+ T‐cells from the COVID‐19 Multi‐omics Blood Atlas Consortium showing the density of UCell score for ISG signature. Data representative of 80 samples. (D) Boxplot showing frequency of ISG cluster by disease type per donor. (n) value indicates the number of unique donors. p‐value calculated using a two‐tailed t‐test. ns = p > 0.05, *p < 0.05, **p < 0.01, ***p < 0.001, ****p < 0.0001 [file EJI-55-e202451371-s002.pdf]
